# Determinants of health care seeking behaviour during pregnancy in Ogun State, Nigeria

**DOI:** 10.1186/s12978-016-0139-7

**Published:** 2016-06-08

**Authors:** David O. Akeju, Olufemi T. Oladapo, Marianne Vidler, Adepoju A. Akinmade, Diane Sawchuck, Rahat Qureshi, Muftaut Solarin, Olalekan O. Adetoro, Peter von Dadelszen, and the CLIP Nigeria Feasibility Working Group

**Affiliations:** Department of Sociology, University of Lagos, Lagos, Nigeria; UNDP/UNFPA/UNICEF/WHO/World Bank Special Programme of Research, Development and Research Training in Human Reproduction (HRP), Department of Reproductive Health and Research, World Health Organization, Geneva, Switzerland; Department of Obstetrics and Gynaecology, and the Child and Family Research Unit, University of British Columbia, Vancouver, BC Canada; Centre for Research in Reproductive Health, Sagamu, Ogun State Nigeria; Department of Obstetrics and Gynaecology, Aga Khan University, Karachi, Pakistan; Directorate Division of Medical and Health Care Services, Ijebu Ode Local Government Secretariat, Ijebu Ode, Ogun State Nigeria; Department of Obstetrics and Gynaecology, Olabisi Onabanjo University, Sagamu, Ogun State Nigeria

**Keywords:** Health care, Pregnancy complications

## Abstract

**Background:**

In Nigeria, women too often suffer the consequences of serious obstetric complications that may lead to death. Delay in seeking care (phase I delay) is a recognized contributor to adverse pregnancy outcomes. This qualitative study aimed to describe the health care seeking practices in pregnancy, as well as the socio-cultural factors that influence these actions.

**Methods:**

The study was conducted in Ogun State, in south-western Nigeria. Data were collected through focus group discussions with pregnant women, recently pregnant mothers, male decision-makers, opinion leaders, traditional birth attendants, health workers, and health administrators. A thematic analysis approach was used with QSR NVivo version 10.

**Results:**

Findings show that women utilized multiple care givers during pregnancy, with a preference for traditional providers. There was a strong sense of trust in traditional medicine, particularly that provided by traditional birth attendants who are long-term residents in the community. The patriarchal c influenced health-seeking behaviour in pregnancy. Economic factors contributed to the delay in access to appropriate services. There was a consistent concern regarding the cost barrier in accessing health services. The challenges of accessing services were well recognised and these were greater when referral was to a higher level of care which in most cases attracted unaffordable costs.

**Conclusion:**

While the high cost of care is a deterrent to health seeking behaviour, the cost of death of a woman or a child to the family and community is immeasurable. The use of innovative mechanisms for health care financing may be beneficial for women in these communities to reduce the barrier of high cost services. To reduce maternal deaths all stakeholders must be engaged in the process including policy makers, opinion leaders, health care consumers and providers. Underlying socio-cultural factors, such as structure of patriarchy, must also be addressed to sustainably improve maternal health.

**Trial registration:**

NCT01911494

**Electronic supplementary material:**

The online version of this article (doi:10.1186/s12978-016-0139-7) contains supplementary material, which is available to authorized users.

## Background

Annually, thousands of women are faced with pregnancy-related complications, most of which are attributed to haemorrhage, puerperal sepsis, obstructed labour, hypertensive disorders, and unsafe abortions [1]. Over the last three decades, Nigeria continues to have one of the highest maternal mortality ratios, with 496–560 deaths per 100,000 live births [2, 3]. This high mortality ratio is accompanied by high fertility rates, increasing the obstetric risk for Nigerian women [4]. Women are at further risk of morbidity and mortality due to poor health seeking practices and limited access to health facilities. There is a gross deficiency in the distribution of health facilities; many communities in rural Nigeria do not have good access to facilities staffed with qualified personnel [5]. This insufficient number of facilities may partially account for the low rate of institutional deliveries [4]. Furthermore, roads are often inaccessible and transportation systems are problematic [5, 6].

Others studies have also shown that the use of health care services is related to the availability, quality and cost of services, as well as to the social structure, health beliefs and personal characteristics of the users [7, 8]. Cost affects health care behaviour significantly as do other socio-demographic factors such as occupation, parity, education, maternal age, and distance to health facility [9, 10]. Comprehensive antenatal care (ANC) has the capacity to reduce maternal mortality; however, many do not have the ability to pay for such services [11, 12]. In addition, a study conducted among women in a south-eastern part of Nigeria showed that women usually report late for ANC due to the belief that there are no advantages in early booking, as ANC is perceived primarily as curative rather than preventive [13].

Some studies have shown that health-related knowledge does not translate to increased utilization of services in pregnancy [14, 15]. In Nigeria, the ability of women to seek care is significantly moderated by the cost of antenatal care [12]. Understanding social and cultural factors that influence health care seeking behaviour is critical for ensuring safe pregnancies and deliveries. This study builds on previous work by investigating the influence of community factors on a woman’s decision to seek care during pregnancy in Ogun State, Nigeria.

## Methods

### Study site

**Fig. 1:**
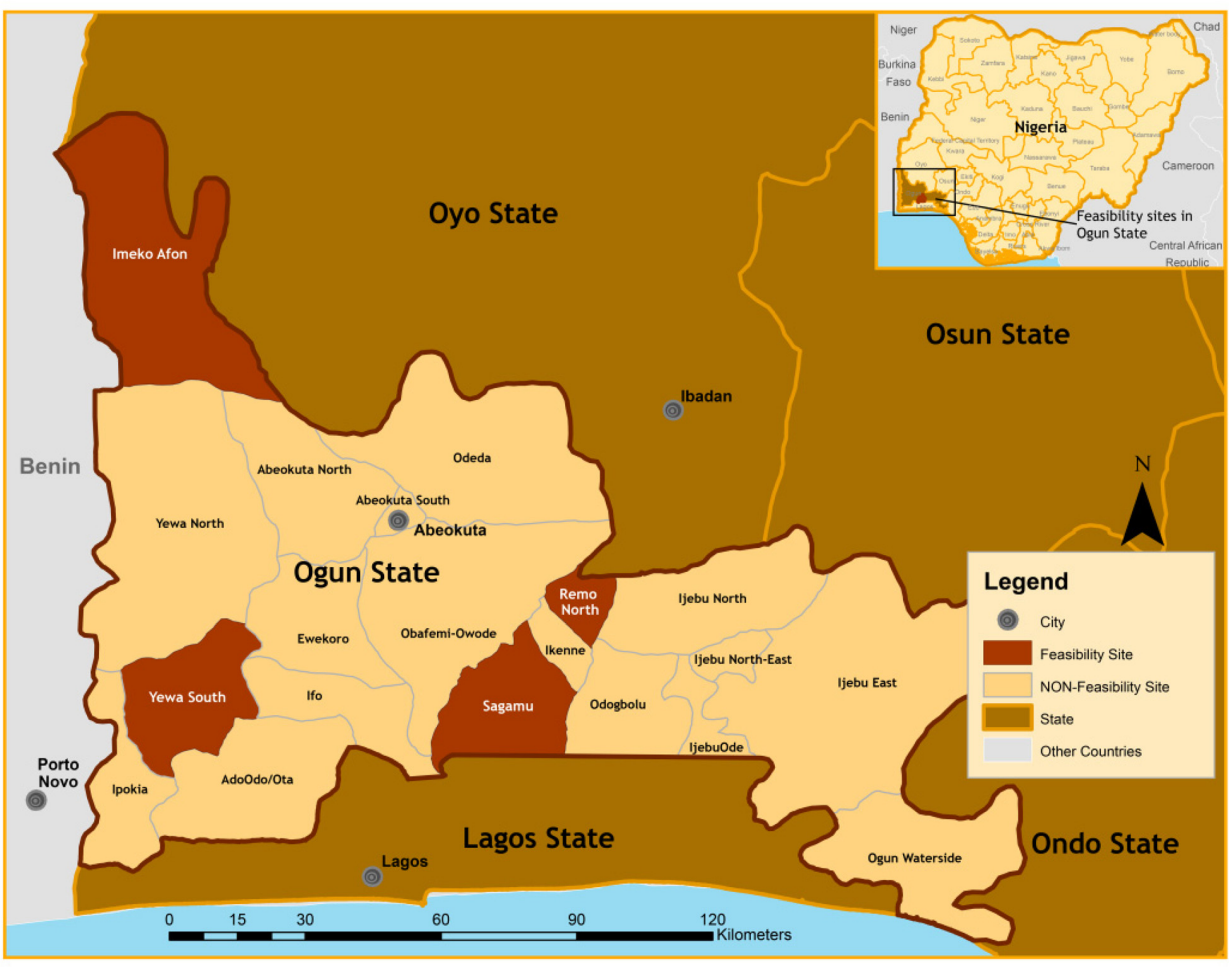
Map of study sites

The study was conducted in four Local Government Areas in Ogun State, Nigeria: Ogijo, Yewa South, Imeko-Afon and Remo North (Table 1 and Fig. 1). Ogun State is one of 36 states and is located in the southwest geo-political zone. It covers a total land area of 16,409 km^2^. It has a projected population of 4.3 million people and the predominant ethnic group is Yoruba. Farming is the main occupation, largely subsistence farming and cash crops of cocoa and kolanut. In the urban and suburban areas, petty trading and blue-collar jobs are the major occupation.

**Table 1:** Study site characteristics

Nigeria characteristics	
Population	159,288,426
Size (Km^2^)	923, 768
Number of states	36
Number of geopolitical zones	6
Predominant language	Yoruba, Igbo, and Hausa
Predominant religions	Christianity and Islam
Ogun State characteristics	
Population	4,000,000
Size (Km^2^)	16,409
Number of local government areas	20
Predominant language	Yoruba
Predominant religion	Christianity
Local Government Area characteristics	
Cumulative population	469,271
Cumulative size (Km^2^)	1657
Number of study areas	4/40

### Study design

This study is part of a larger initiative aimed at assessing community level interventions for the management of pre-eclampsia and eclampsia in Nigeria. An ethnographic framework was used to gain insight into the social and cultural realities at the community level, as these are thought to influence health seeking behaviours.

Data was collected through focus group discussions with pregnant women, recently pregnant mothers, male decision-makers, opinion leaders, traditional birth attendants (TBAs), community health extension workers (CHEWs), nurses and midwives. In addition, interviews were held with local administrative personnel, private medical practitioners, head TBAs, head CHEWs, chief nursing officers, chief medical directors, medical officers, and community leaders (Tables 2 and 3). Each focus group discussion and interview was audio-recorded and transcribed. Data analysis was carried out using NVivo version 10.

**Table 2:** Focus group discussion characteristics

Number	N participants	Region	Age (yr) Median [range]	Religion 1 Islam 2 Christian 3 Traditional religion	N children Median [range]	% Married
Community/Opinion Leaders						
1	12	Yewa South	52 [27,70]	1 = (*N* = 6) 2 = (*N* = 6)	5 [0,6]	100 %
2	10	Remo North	44 [43,77]	1 = (*N* = 3) 2 = (*N* = 5) 3 = (*N* = 2)	*Not known*	100 %
3	12	Remo North	58 [30,85]	*Not known*	*Not known*	100 %
4	10	Ogijo	50 [26,71]	1 = (*N* = 5) 2 = (*N* = 5)	6 [1, 9]	100 %
5	12	Ogijo	57 [45, 72]	1 = (*N* = 3) 2 = (*N* = 6) 3 = (*N* = 3)	8 [4, 10]	100 %
6	12	Imeko-Afon	45 [20,55]	1 = (*N* = 6) 2 = (*N* = 6)	3 [0,10]	100 %
Male Decision-Makers						
1	12	Yewa South	38 [27,49]	1 = (*N* = 2) 2 = (*N* = 10)	3 [1, 5]	100 %
2	11	Remo North	40 [35,62]	1 = (*N* = 4) 2 = (*N* = 6) 3 = (*N* = 1)	3 [0,9]	100 %
3	12	Imeko-Afon	51 [40,60]	1 = (*N* = 11) 2 = (*N* = 1)	6 [4, 10]	100 %
Recently Pregnant Mothers						
1	12	Yewa South	27 [20,42]	1 = (*N* = 6) 2 = (*N* = 6)	3 [1, 5]	100 %
2	12	Yewa South	31 [20,42]	1 = (*N* = 4) 2 = (*N* = 8)	2 [1, 3]	100 %
3	12	Remo North	29 [21,39]	1 = (*N* = 3) 2 = (*N* = 9)	4 [1, 6]	100 %
4	12	Remo North	28 [21,34]	1 = (*N* = 5) 2 = (*N* = 7)	2 [1, 4]	100 %
5	12	Ogijo	31 [26,43]	1 = (*N* = 4) 2 = (*N* = 8)	3 [1, 4]	92 %
6	12	Ogijo	29 [22,38]	1 = (*N* = 1) 2 = (*N* = 11)	2 [1, 5]	100 %
7	12	Imeko-Afon	30 [16,36]	1 = (*N* = 5) 2 = (*N* = 7)	3 [1, 5]	100 %
8	11	Imeko-Afon	30 [18,36]	1 = (*N* = 6) 2 = (*N* = 5)	3 [1, 6]	100 %
Pregnant Women						
1	12	Yewa South	26 [20,33]	1 = (*N* = 3) 2 = (*N* = 9)	1 [0,4]	100 %
2	12	Yewa South	26 [20,39]	1 = (*N* = 4) 2 = (*N* = 8)	3 [1, 3]	100 %
3	12	Remo North	30 [20,36]	1 = (*N* = 1) 2 = (*N* = 11)	1 [1, 3]	100 %
4	12	Remo North	32 [23,40]	1 = (*N* = 5) 2 = (*N* = 7)	3 [1, 5]	100 %
5	9	Ogijo	27 [19,34]	1 = (*N* = 6) 2 = (*N* = 3)	1 [0,2]	100 %
6	10	Imeko-Afon	22 [19,26]	1 = (*N* = 7) 2 = (*N* = 3)	1 [0,4]	100 %
7	12	Imeko-Afon	25 [20,30]	1 = (*N* = 2) 2 = (*N* = 10)	2 [0,4]	100 %
Traditional Birth Attendants						
1	12	Yewa South	44 [32,65]	1 = (*N* = 7) 1 = (*N* = 5)	3 [1,4]	100 %
2	12	Remo North	50 [41,77]	1 = (*N* = 1) 2 = (*N* = 8) 3 = (*N* = 2)	5 [3,5]	100 %
3	12	Ogijo	40 [25,50]	1 = (*N* = 5) 2 = (*N* = 6)	4 [0,5]	83 %
Community Health Extension Workers						
1	12	Yewa South	40 [28,53]	1 = (*N* = 5) 2 = (*N* = 7)	3 [0,4]	100 %
2	12	Yewa South	32 [28,55]	2 = (N12)	1 [0,4]	100 %
3	12	Ogijo	38 [35,51]	1 = (*N* = 2) 2 = (*N* = 10)	3 [2,5]	100 %
4	12	Ogijo	39 [33,50]	1 = (*N* = 4) 2 = (*N* = 8)	2 [2,5]	100 %
5	11	Remo North	38 [32,50]	2 = (*N* = 11)	2 [0,5]	100 %
Nurses and Midwives						
1	10	Ogijo	50 [30,53]	1 = (*N* = 2) 2 = (*N* = 8)	3 [1,4]	80 %
2	12	Yewa South	49 [46,54]	1 = (*N* = 2) 2 = (*N* = 8)	3 [2,6]	83 %
3	9	Remo North	46 [32,55]	1 = (*N* = 1) 2 = (*N* = 8)	3 [0,4]	78 %

**Table 3:** Interview characteristics

Number	Stakeholder Group	Cluster
1	Head of Traditional Birth Attendants	Sagamu
2	Head of Traditional Birth Attendants	Yewa South
3	Head of Traditional Birth Attendants	Imeko-Afon
4	Head of Traditional Birth Attendants	Remo North
5	Community Leader	Imeko-Afon
6	Male Community Leaders	Imeko-Afon
7	Women Community Leaders	Sagamu
8	Women Community Leaders	Imeko-Afon
9	Women Community Leaders	Remo North
10	Opinion Leaders	Yewa South
11	Opinion Leaders	Remo North
12	Opinion Leaders	Imeko Afon

The Health Research and Ethics Committee (HREC) of Olabisi Onabanjo University Teaching Hospital, Sagamu, Nigeria (OOUTH/DA/326/431) and the Clinical Research Ethics Board of the University of British Columbia, Vancouver, Canada (H12-00132), approved the study.

## Results

These results identified factors that influence health care seeking behaviour in pregnancy in Ogun. These factors included location, time, obstetric condition and socio-cultural characteristics described below.

### Where women seek care

Respondents reported patronizing multiple types of health care providers in pregnancy. Some women preferred services offered at the health centre or government hospital, however most favoured traditional doctors, prayer houses and TBAs in pregnancy. The use of traditional providers or prayer houses does not reportedly prevent women from registering at the health centre, and it was rare to find women who patronized only one type of provider.

*Pregnant women seek care at various places these days….some would go to a spiritual leader’s place and remain in hibernation…some would go to the herbalist’s place…some would go to the Islamic priest’s place….some would go to “Iya l’osha”….and some would go to the hospital. All these places work…sometimes when a pregnant woman seeks care at the hospital…it might get to a stage where the health care workers would ask the pregnant woman to seek alternative therapies….and the pregnant woman would return home…and she would wait for God to answer her prayers. A pregnant woman could seek help from all these places.* [Opinion and Religious Leaders]

Furthermore, some believed pregnancy complications required spiritual intervention; in these cases women visited “*Iya l’osha”* (a female priest) who is believed to cure complications of supernatural origin. In some cases, ‘*Iya l’osha*’ was used as a last resort when all efforts using orthodox medicine had failed.

Some TBAs reported referring women with complications to the health centre. According to one CHEW, close to 60 % of women patronize TBAs, and when TBAs cannot handle an obstetric complication, they refer women to the health centres.

*About 55–60 % prefer going to the TBA. […] Some women prefer going there and when there is any problem, some TBAs will refer them to health centres.* [Community Health Extension Workers]

It is evident that traditional providers have established themselves in these communities. Women affirmed that their choice to patronize traditional providers was due to the strong interpersonal relationships, reduced cost and the ease of payment. This was well described by a CHEW during one of the discussions:

*The women cannot pay much money at the TBA … they can give them a drug that they have, after the treatment, maybe 3 months after they would collect their money. But in government hospital you cannot allow them to come after 3 months when they’ve received treatment… to come back and pay the money. So because of poverty, they prefer to go to TBA. [*Community Health Extension Workers]

In addition, respondents described a belief that “*hospital medications only reduce the severity of illnesses like malaria”* whereas, “*[a] local concoction cleanses their body of all toxins.”* The perception was generally held that some complications are better treated by traditional doctors. Explaining this point further, a male decision-maker described how women patronized both traditional health care providers and skilled professionals: “*once they detect that the baby is lying across in the belly, they go to traditional doctors for care and they usually change the position of the baby to normal position”.*

While health care seeking behaviour is influenced by the factors mentioned above, delays are influenced by additional factors.

### When women seek care

The general view among the community and health care providers was that women accessed the formal health care system when they perceived they were at risk. Usually, this was for delivery-related care, particularly deliveries complicated by obstructed labour or retained placenta.

*They would come to us when they have complications…after they had gone to seek care at other place […] when they encounter problems at those other places, they would advise them that “you should go to the health centre” and the pregnant woman would just be helpless.* [Community Health Extension Workers]

In addition to obstetric complications which persuade women to seek care, the time of the day can be an important factor in decision-making. Often, health care facilities are closed at night due to human resource constraints; this unavailability causes women to deliver with traditional providers. A new mother gave an account that portrays this point.

*It wasn’t that I wanted to deliver at the herbalist’s place…initially, we went to a State Hospital…like around 11:30 p.m. or about some minutes to 12:00 midnight....the place was closed. We knocked and knocked on the door…in the middle of the night…no one answered. […] I was registered with the State hospital […] I left there and decided to seek care from the herbalist because there was no other place for us to go to seek care.* [New Mother]

Frequent health facility closures are worsened by the significant distance, poor road access, and unavailability of transport late at night. In some cases, women preferred to patronize traditional birth attendants, rather than travel long distances to seek care with qualified health personnel.

### Cultural factors

The community indicated that women delay revealing their pregnancies as long as possible. It was believed that early disclosure may lead to miscarriage and other complications. This belief was connected to the notion that supernatural and diabolic forces had the potential to influence pregnancy outcomes. As a result , routine activities that could reveal pregnancy status, including antenatal care, are reportedly avoided. Respondents claimed that it was common practice for women to delay antenatal care until the seventh month of pregnancy. Some women chose to deliver with TBAs, as they associated hospitals with surgical interventions, and there was a strong cultural preference for ‘unassisted deliveries’.

*She was using a hospital. When it was time for her, they tested her and told her that her womb wasn’t wide enough for her to deliver the baby herself that she had to undergo a surgical operation…the moment she heard the word surgical operation instead of going to the hospital for treatment, she went to the herbalist’s place. She labored for a long time at the herbalist’s place and later, the herbalist asked her to leave when he saw that he couldn’t handle the situation. She then went to a hospital, but the hospital rejected her….and she died before she could get to the General Hospital.* [Male Decision-Maker]

In addition to the fears of surgery, some also expressed fear of other hospital interventions, such as the use of ultrasound.

*It is the scan result that makes some pregnant women go to the herbalist’s place to seek treatment. […] Health care workers could tell some pregnant women that their babies is lying in awkward position…..and they might need to turn their babies to the right position.....this is what make many pregnant women to go to the herbalist’s place to seek care.* [New Mother]

To some extent, these cultural norms were influenced by gender relation and the culture of patriarchy that predominates in Nigeria. The study areas had a dominant culture of patriarchy, and the man’s consent played a significant role in determining where and when a woman could seek health care. Discussions with health workers showed that some women would not start ANC until they were permitted to do so by their husband. Women who made independent health care decisions were considered to be arrogant, disrespectful and in the word of one female participant, “*too forward”*.

*If the woman doesn’t seek the consent of the husband before deciding where to seek care, the woman will be considered as being too forward.* [Male Decision-Maker]

### Financial constraints and cost of services

Women delayed care seeking due to financial constraints; women depended mainly on their husbands to supply funds for health services. In cases where the husband was unable to pay, family members or friends may have covered the costs.

*Some of them might say that their spouses have not given them the go-ahead to do so even if they have the money to pay. It is when the husband has money that she can come she cannot take the decision on her own.* [Community Health Extension Worker]

Finances were a consistent barrier to health care services for many, and this barrier was greatest for higher-level care. Teenage mothers were felt to be at particularly high risk as they were less likely to be financially prepared. Reportedly, pregnant women tended to save in the event that it is required during pregnancy. They tended to raise funds near delivery when costs were likely to be incurred. It was perceived to be essential to have funds protected in advance as it was uncertain what might happen during pregnancy.

*Pregnant women should not be tight fisted, if the husband refuses to give money; the wife also needs to raise money for herself.* [Pregnant Woman]

To raise funds, family members sometimes sold property. In rare cases, health workers contributed when women were unable to pay. As such, the cost of services greatly influenced the choice of provider and facility. Cost also influenced the ability of women to follow through with referrals.

*I was sick during pregnancy and I went to the State Hospital, they wrote me a bill of over four thousand Naira when they hadn’t even given me a single medication. They didn’t even offer me paracetamol to use, they just abandoned me on the bed. My husband was running around to raise money.* [New Mother]

Women patronize the herbalist, church or TBA because they allow delayed payment. According to a TBA “*They also choose us because it is more expensive to go to the hospital or health centres around here.”* These outlets operated on different modes of payment that enabled women to pay in instalments and with other forms of payment such as goats or palm oil. This made it easier for women to facilitate payment for health care with TBAs.

## Discussion

These findings demonstrate that women commonly utilize several obstetric health care providers in complement during pregnancy: traditional birth attendants, faith-based providers, and orthodox practitioners (nurses, midwives, community health workers, doctors, specialists). As described by a sample of community members, health care decisions in pregnancy are influenced by cultural norms and beliefs, perceived quality of care, time of day, cost of services, and transport options.

Health care decisions in pregnancy can be explained by use of the Health Belief Model, which states that individuals weigh the potential benefits against the psychological, physical and financial costs when making decision to seek care, as was reflected in these findings [16]. In most African societies, the status of women is low [17] and families are patriarchal, with men responsible for key decisions. The patriarchal family structure rests on men's control over property; this often extends to the wife as his possession. It is within this cultural milieu of male-dominance that women enter into marriage, child bearing and child rearing in Nigeria. The patriarchal culture gives women little or no power to decide when they become pregnant, or how, when, and where to seek care during complications [17]. While patriarchy is a culturally entrenched factor that subjugates  women , lack of financial empowerment further compounds women’s inabilities to determine where, when and how they may seek care during pregnancy. Thus the inability of most Nigerian women to pay for maternal health services drives them to alternatives such as the use of local herbs and consultation with traditional birth attendants and doctors. Government hospitals are usually patronized when all alternatives have been exhausted. Against the backdrop of endemic poverty, women are constrained from seeking care at the health centre or government hospital. As observed in a recent study [18], the cost of transport is an additional cost to care seeking. Distance to health facility is unequal, greatest in rural areas and tends to escalate the cost of care.

The strength of this study lies in its adoption of an ethnographic framework, enriched by real life experiences and realities of health care providers and patients. Although the study has been able to describe some determinants of health care seeking behaviour, a limitation is that we cannot infer causation of these factors and the observed outcomes beyond the study sites. It is expected that similar socio-cultural factors underpin health care decision making in other rural African communities; however, due to the qualitative nature of this study generalizability cannot be presumed.

It is important for the Nigerian government to reduce financial barriers by decreasing the cost or providing free health services in pregnancy. Although the provision of free health care services was reported in Ogun State, this change has not reached all areas of the state. In addition, targeted health messaging should be provided to reduce knowledge gaps, empower pregnant women and engage community leaders. These findings should be used to guide health messaging to increase utilization of maternal health care services. It is evident that such educational approaches must dispel harmful myths surrounding pregnancy, such as the effect of early pregnancy disclosure, as well as highlighting the importance of facility-based care to improve maternal outcomes. These public health messages should also include important health system information, such as facility location and hours. The findings of this study have been presented to key local stakeholder groups through meetings which included local media coverage. Attendants at these events included pregnant women and families, community-based health care providers, and current and past government representatives at the local and state level.

## Conclusion

A community participatory approach is needed to co-ordinate maternal health services in Ogun State and Nigeria at large. Efforts should be made towards community engagement and education to better integrate various providers and community stakeholders, including male decision-makers. Educational messaging should highlight the importance of appropriate and timely access to health care services. Cost of care must also be addressed; one option is incorporating a community financing scheme into the National Health Insurance Scheme. There is of course no magic bullet; all solutions are long-term approaches which require considerable financial investment.

### Peer review

Peer review reports for this article can be found in Additional file 1.

## Additional file

Additional file 1: Peer review reports. (PDF 329 kb)

## Abbreviations

ANC: antenatal care
CHEWs: community health extension workers
TBAs: traditional birth attendants
