# Supplementary material for: Determinants of health care seeking behaviour during pregnancy in Ogun State, Nigeria
Source: Reprod Health. 2016 Jun 8;13(Suppl 1):32. doi: 10.1186/s12978-016-0139-7 (PMC4943510; doi:10.1186/s12978-016-0139-7)
Supplement: Additional file 1: — Peer review reports. (PDF 329 kb) [file 12978_2016_139_MOESM1_ESM.pdf]

Referee's comments to the authors– this sheet WILL be seen by the author(s) and published with the article

Title	**Health Care Seeking for Pregnancy Complications in Ogun State, Nigeria**
Author(s)	David O Akeju, Olufemi T. Oladapo, Marianne Vidler, Akinmade O Adepoju, Diane Sawchuck, Rahat Qureshi, Muftaut Solarin, Olalekan O. Adetoro, Peter von Dadelszen and the CLIP Nigeria Feasibility Working Group
Referee's name	Mandisa Singata -Madliki

When assessing the work, please consider the following points, where applicable:

1. Is the question posed by the authors new and well defined?
2. Are the methods appropriate and well described, and are sufficient details provided to replicate the work?
3. Are the data sound and well controlled?
4. Does the manuscript adhere to the relevant standards for reporting and data deposition?
5. Are the discussion and conclusions well balanced and adequately supported by the data?
6. Do the title and abstract accurately convey what has been found?
7. Is the writing acceptable?

Please make your report as constructive and detailed as possible in your comments so that authors have the opportunity to overcome any serious deficiencies that you find and please also divide your comments into the following categories:

- Major Compulsory Revisions (which the author must respond to before a decision on publication can be reached)
- Minor Essential Revisions (such as missing labels on figures, or the wrong use of a term, which the author can be trusted to correct)
- Discretionary Revisions (which are recommendations for improvement but which the author can choose to ignore)

Where possible please supply references to substantiate your comments.

When referring to the manuscript please provide specific page and paragraph citations where appropriate.

General comments:

Thank you for the invitation to review this paper on Health care seeking for pregnancy complications on Ogun State,Nigeria.There are several intriguing findings and opportunities for further investigation, as discussed by authors.

The authors need to be congratulated for their work as this is an important issue in public health, given the morbidity and mortality associated with unmanaged pregnancy complications.

Major compulsory revisions:
None

Discretionary revisions:

Authors have already made some recommendations in the conclusions ,what will be more interesting is to share their plan of sharing these findings to the local policy makers as some of the factors that inhibit early access to health care are related to existing health policies ,to mention but few pregnant women need to pay for health care.

(continue on the next sheet)

Continued:

One of the central findings is the control of males over when women become pregnant and the care they receive. It would have been interesting to include discussion with the informants on the control of contraceptive decision-making.

It would be useful to have more discussion on specific public health measure which might be used to address the constraints on health care seeking identified.

Further discussion of the generalizability (or lack of generalizability) of the findings elsewhere in Nigeria as well as globally would be useful.

Line 255-6, there seems to be some text missing.

Referee's comments to the authors– this sheet WILL be seen by the author(s) and published with the article

Title	**Health Care Seeking for Pregnancy Complications in Ogun State, Nigeria**
Author(s)	David O Akeju, Olufemi T. Oladapo, Marianne Vidler, Akinmade O Adepoju, Diane Sawchuck, Rahat Qureshi, Muftaut Solarin, Olalekan O. Adetoro, Peter von Dadelszen and the CLIP Nigeria Feasibility Working Group
Referee's name	Nelson Sass

When assessing the work, please consider the following points, where applicable:

1. Is the question posed by the authors new and well defined?
2. Are the methods appropriate and well described, and are sufficient details provided to replicate the work?
3. Are the data sound and well controlled?
4. Does the manuscript adhere to the relevant standards for reporting and data deposition?
5. Are the discussion and conclusions well balanced and adequately supported by the data?
6. Do the title and abstract accurately convey what has been found?
7. Is the writing acceptable?

Please make your report as constructive and detailed as possible in your comments so that authors have the opportunity to overcome any serious deficiencies that you find and please also divide your comments into the following categories:

- Major Compulsory Revisions (which the author must respond to before a decision on publication can be reached)
- Minor Essential Revisions (such as missing labels on figures, or the wrong use of a term, which the author can be trusted to correct)
- Discretionary Revisions (which are recommendations for improvement but which the author can choose to ignore)

Where possible please supply references to substantiate your comments.

When referring to the manuscript please provide specific page and paragraph citations where appropriate.

General comments: The article is a picture of the local condition and, under a multidisciplinary approach, tries to explain some reasons related to high numbers of maternal deaths in the region. The authors highlight cultural patterns, political and economic reality in the place as possible factors associated with maternal and perinatal outcomes. These conditions are not unique to this community because the association between poverty, low educational degree and the absence of a strong health policy results in very worrying health indicators. I think that the title would be changed because the article try to describe conditions related to difficulties to reach health care (health care seeking behavior).

Despite of the authors' suggestion that community health financing scheme incorporated into the National Health Insurance scheme would be important to improve the conditions, the actual situation wouldn't be modified immediately. Strategies to empower opinion leaders have to be considered in order to qualify the care of these women. However, cultural and social patterns are not changed immediately.

From the information registered, the authors point no accurate measurement related to outcomes, but it is hard to believe that such situation is not related to maternal and perinatal deaths. Perhaps the discussion could incorporate some specific propositions along with community leaders, especially among male community, in order to highlight the importance of early treatment and preservation of families.

Finally, the authors offer a very accurate diagnosis of the reasons related to those observed outcomes, but should be considered that the implementation of modification factors requires time and your success will depend on factors difficult to control, at least in the short term.

Major compulsory revisions:

Minor essential revisions:

A checklist over the references is required in order to reach Vancouver standard. As an example, note the differences in the reference number 14. In the text it is recorded as follows:

GO Akaba, JAM Otubu, ET Agida, O Onafowokan. Knowledge and utilization of malaria preventive measures among pregnant women in FCT, Nigeria. Nigerian Journal of Clinical Practice, Apr-Jun 2013, Vol 16, Issue 2 pg 201-206.

However, In PubMed we can see the follow:

GO Akaba, JAM Otubu1 , ET Agida, O Onafowokan. Knowledge and utilization of malaria preventive measures among pregnant women at a tertiary hospital in Nigeria's federal capital territory. Niger J Clin Pract. 2013; 16(2):201-6.

Discretionary revisions:

Supplement Editor's comments:

Please do not make comments that are beyond the results of your study. Please remove or modify the first sentence of the abstract .

I wonder if you can do a better organization of the Discussion section. It is useful to begin the discussion by briefly summarizing the main findings, then explore possible mechanisms or explanations for these findings, compare and contrast the results with other relevant studies, state the limitations of the study, and explore the implications of the findings for future research and for clinical practice.
